# Cognition in Context: Understanding the Everyday Predictors of Cognitive Performance in a New Era of Measurement

**DOI:** 10.2196/14328

**Published:** 2020-07-23

**Authors:** Emma Weizenbaum, John Torous, Daniel Fulford

**Affiliations:** 1 Department of Psychological and Brain Sciences Boston University Boston, MA United States; 2 Department of Psychiatry Beth Israel Deaconess Medical Center Harvard Medical School Boston, MA United States; 3 Department of Occupational Therapy and Rehabilitation Sciences Boston University Boston, MA United States

**Keywords:** smartphone, mobile phone, neuropsychology, individualized medicine

## Abstract

**Background:**

Research suggests that variability in attention and working memory scores, as seen across time points, may be a sensitive indicator of impairment compared with a singular score at one point in time. Given that fluctuation in cognitive performance is a meaningful metric of real-world function and trajectory, it is valuable to understand the internal state-based and environmental factors that could be driving these fluctuations in performance.

**Objective:**

In this viewpoint, we argue for the use of repeated mobile assessment as a way to better understand how context shapes moment-to-moment cognitive performance. To elucidate potential factors that give rise to intraindividual variability, we highlight existing literature that has linked both internal and external modifying variables to a number of cognitive domains. We identify ways in which these variables could be measured using mobile assessment to capture them in ecologically meaningful settings (ie, in daily life). Finally, we describe a number of studies that have already begun to use mobile assessment to measure changes in real time cognitive performance in people’s daily environments and the ways in which this burgeoning methodology may continue to advance the field.

**Methods:**

This paper describes selected literature on contextual factors that examined how experimentally induced or self-reported contextual variables (ie, affect, motivation, time of day, environmental noise, physical activity, and social activity) related to tests of cognitive performance. We also selected papers that used mobile assessment of cognition; these papers were chosen for their use of high-frequency time-series measurement of cognition using a mobile device.

**Results:**

Upon review of the relevant literature, it is evident that contextual factors have the potential to meaningfully impact cognitive performance when measured in laboratory and daily life environments. Although this research has shed light on the question of what gives rise to real-life variability in cognitive function (eg, affect and activity), many of the studies were limited by traditional methods of data collection (eg, involving retrospective recall). Furthermore, cognition has often been measured in one domain or in one age group, which does not allow us to extrapolate results to other cognitive domains and across the life span. On the basis of the literature reviewed, mobile assessment of cognition shows high levels of feasibility and validity and could be a useful method for capturing individual cognitive variability in real-world contexts via passive and active measures.

**Conclusions:**

We propose that, through the use of mobile assessment, there is an opportunity to combine multiple sources of contextual and cognitive data. These data have the potential to provide individualized digital signatures that could improve diagnostic precision and lead to meaningful clinical outcomes in a wide range of psychiatric and neurological disorders.

## Introduction

### Background

Thinking occurs dynamically. From day to day and moment to moment, our ability to hold information in mind or regulate attention is constantly in a state of flux. The sources of these fluctuations are varied—whether coming in the wake of mid-afternoon sleepiness, an anxiety-provoking presentation, or the confusion of navigating a crowded store, cognitive performance is influenced by the contexts in which a person operates. Despite this inherent variability, researchers and clinicians often assume that cognitive performance reflects internal processes that are constant and stable. This assumption leads to the characterizations of cognitive performance as static, treating the *mean* of an individual’s or group’s cognitive performance as the output of internal processes and dismissing variance in performance as *noise*. Research has found that these within-person changes in performance, commonly referred to as *intraindividual cognitive variability* [1,2], can serve as highly sensitive markers of cognitive dysfunction [3], such as in attention deficit hyperactivity disorder [4] and cognitive decline [5] or dementia [6,7]. Intraindividual variability has been used to describe change or dispersion across domains of function (eg, memory vs attention) [8], variability or inconsistency in response within a single measurement time point [9], or across a number of time points of varying scales (eg, moment-to-moment fluctuations) [10]. In this paper, we seek to identify the factors that give rise to changes in attention and working memory (given these domains’ susceptibility to fluctuation) [11] *within individuals*, *across time* (eg, 1-2 weeks) and *contexts* (eg, physical environment and emotional state).

To effectively measure how contexts shape attention and working memory, dense sampling of cognitive performance across time and in various settings is essential. Measuring performance across a series of time points in traditional laboratory or clinic settings is arduous and expensive and represents a limited and unnatural context for cognitive function. Instead, by using an experience sampling method (ESM) or ecological momentary assessment (EMA) of frequent testing over the course of days or weeks [12], high-resolution measurements can be obtained, which give rise to a rich picture of the dynamics and patterns of an individual’s cognitive functioning within a period of time [13]. Until recently, methods of studying frequency and patterns of behavior in daily life relied on pen-and-paper responses or frequent calls to participants that were burdensome and intrusive. However, the increasing ubiquity of mobile devices (eg, smartphones and tablets) allows for the feasible acquisition of high-frequency data [14,15], which can be collected in vivo, without the limitations of retrospective reporting. Mobile assessment data, in turn, produce a *digital phenotype* [16] (ie, a reflection of a person’s moment-to-moment cognitive and behavioral function in the context of everyday life). The goal of digital phenotyping aligns with the advancement of precision medicine, where personalized models predict function based on relevant internal and environmental variables. The use of this method has become increasingly common in the fields of clinical psychology and psychiatry to understand the temporal patterns of symptoms [17,18] and how contextual factors, such as a person’s physical and social environment, influence important clinical features, such as mood dysregulation [19]. However, digital phenotyping is just beginning to be validated within the field of neuropsychology as digital cognitive assessments are being developed and implemented [15,20,21].

### Objective

On the basis of the research available to date, we propose that significant strides could be made in predicting meaningful health outcomes by assessing the everyday factors that influence cognition via smartphones or other mobile technologies. In this viewpoint, we begin by describing the importance of measuring intraindividual cognitive variability, focusing on the domains of attention and working memory. To better understand the factors that give rise to this variability, we explore how internal state-based variables (eg, affect and motivation) and external contextual variables (eg, time of day, surrounding noise, and activity) affect cognitive performance. Finally, we propose using mobile assessment as a means of integrating ambulatory cognitive assessment tasks with contextual and environmental data to produce digital phenotypes that could aid in diagnostic precision.

### Intraindividual Variability in Cognition

Intraindividual variability can be observed at multiple time scales [1], and yet, the majority of literature has focused on trial-to-trial response variability within a single time point [11,22-25]. However, there is some evidence to suggest that intraindividual variability within and across days is positively correlated, signaling potentially similar processes, although the extent of this generalization is unclear [26]. Broadly defined, variability in attention and inhibition of behavioral response is thought to be supported by the prefrontal cortex, which serves to differentially activate the strength of specific networks depending on factors of context, state, and task demands [23]. One study found the regulation of task performance and adaptive modulation of attention to be localized to the right dorsolateral frontal regions that exert top-down attentional control [22]. Cognitive variability (ie, performance across course of a day) in attention and memory is known to increase in late adulthood [9,27]. Although the function and mechanisms of this increase are not fully understood [11], it seems to occur in both normal aging and varying disease processes [28], resulting in decreased frontal-executive function [29] and an observed decrease in dopamine response within extrastriatal regions [30]. Given that a non-negligible amount of response variability can occur from one time point to another [26], this may limit the generalizability of scores created from aggregate measures that are interpreted to reflect global functioning across many time points [31]. Instead of dismissing the error or noise in these estimates, it is valuable to understand contributors that give rise to the variability seen within one’s performance, specifically how internal state-based and environmental factors shape one’s cognitive performance from moment to moment.

### Modifying Factors of Moment-to-Moment Cognition

Traditional approaches to measuring cognition seek to remove *noise*, which might impact an individual’s performance, such as time of day and distractors in the physical environment. However, in the context of daily life, these factors likely exert meaningful influence on one’s overall function. With the emergence of mobile technology, we now have the opportunity to measure these factors in real time and quantify their influence on cognitive function. As there are only few prior studies that measure the influence of contextual factors on repeat cognitive performance [32-36], whether these factors produce reliable and meaningful patterns of variability remains equivocal. If consistent patterns of within-person variability at either the nomothetic or idiographic level are evident, this could signal opportunities for uniquely tailored real-time intervention points. The potential modifiers of cognitive performance that we will focus on in the following section include internal state-based factors (eg, affect and motivation) and external contextually based factors (eg, time of day, surrounding noise, and recent activity level; Table 1). In addition, we will discuss how each of these modifiers could be more optimally measured in relation to cognitive performance using mobile assessment.

**Table 1:** Internal and external modifiers of cognitive performance.

Reference	Modifier	Sample	Test location and task domain	Length	Result
Jefferies et al [37]	Mood	100 younger adults	In laboratory; visual attention	1 day	Low arousal with negative affect associated with best performance
Ellis et al [38]	Mood	160 younger adults	In laboratory; semantic recall	1 day	Depressed mood reduced semantic processing, interaction between depressed mood and task difficulty
Brose et al [39]	Mood and motivation	101 younger adults	In laboratory; working memory	100 days	Negative affect and low task motivation reduced working memory performance
Brose et al [40]	Mood and motivation	101 younger adults	In laboratory; working memory	100 days	Positive affect and high task motivation improved working memory performance
Sliwinski et al [41]	Mood	184 younger and older adults	In laboratory; working memory	1-2 weeks	Higher stress ratings associated with slower response time on working memory tasks, greater effect in older adults
Salthouse and Berish [36]	Mood	420 adults	Palm-pilot; reaction time	7 days	No relation between mood and reaction time scores
Krawczyk [42]	Motivation	16 younger adults	In scanner; working memory	1 day	Modulation of reward potential correlates with response time and functional magnetic resonance imaging blood oxygen level–dependent response
Yeo and Neal [43]	Motivation	99 younger adults	In laboratory; executive function	1 day	Motivation had the strongest influence on multistep task performance once task was learned and familiar
van der Heijden et al [44]	Time of day	2167 children	In laboratory; sustained attention	1 day	Performance on sustained attention slower but more accurate in the morning
Manly et al [45]	Time of day	10 younger adults	At home; sustained attention	4 days	Positive correlation between errors and sleepiness rating
West et al [46]	Time of day	40 younger and older adults	In laboratory; computer task	4 days	Younger adults performed best on working memory tasks in the evening, older adults performed best in the morning
Lange [47]	Noise	34 younger adults	In laboratory; working memory	1 day	Noise disrupted verbal but not visuospatial working memory performance
Bell and Buchner [48]	Noise	182 younger and 193 older adults	In laboratory; working memory	1 day	Same level of impairment on visual working memory from noise versus silence in younger and older adults
Ljungberg and Neely [49]	Noise	24 adults	In laboratory; reasoning and working memory	1 day	No significant effect of noise on performance, but higher levels of subjective task difficulty and stress ratings
Sibley and Beilock [48]	Activity	48 younger adults	In laboratory; working memory	2 days	Cardiovascular exercise significantly improves working memory
Whitbourne et al [50]	Activity	59 younger adults	Daily diary; subjective complaints	8 days	Older adults report fewer memory failures on days with exercise
Phillips et al [51]	Activity	51 older adults	In laboratory; reasoning and processing speed tasks	5 days	Physical activity accounted for significant within-person variance in cognition, especially processing speed
Allard et al [32]	Activity	60 older adults	Personal digital assistant; semantic memory task	7 days	Intellectually stimulating activities improved semantic memory performance measured later on the same-day
Bielak et al [35]	Activity	146 older adults	Web-based; processing speed, memory, and reasoning	7 days	Faster memory and processing speed on days with individual or small group social activities

### Internal State-Driven Modifiers of Cognitive Performance

In this section, we will cover how a handful of previous studies have measured individuals’ state affect, either through self-report or experimental manipulation, and how it relates to cognitive performance on specific tasks of attention, working memory, and recall. We then examine how momentary motivation, measured either from self-report or via reward manipulations, influences how well people perform on tasks of decision making and working memory. The studies described in this section are listed in Table 1.

#### Affect

The valence (ie, positive or negative) of one’s momentary affect has been shown to impact cognitive performance in the domains of attention, working memory, and recall. Seminal work by Ellis et al [38] demonstrated that when negative affect was induced in healthy undergraduates, using self-referential negative statements, word recall was reduced compared with those in a neutral affect condition, particularly in challenging trials. The authors proposed that in states where strong affect is present, attention is diverted and fewer cognitive resources are allocated for the task at hand; the effects of this become most apparent on difficult tasks that require greater cognitive resources [38]. A separate study of cognition and affect found that visual attention (target detection) varied in relation to the combination of level of arousal and affect valence in a sample of healthy adults. The primary finding was that low arousal-negative affect (eg, sadness) was associated with greater accuracy on second but not the first target detection, indicating that sadness enhanced attention prioritization but not overall improvement of attention [37]; this could be because affect serves to shape the strength of attentional control or allocation [22].

In a study in which self-reported affect and working memory were assessed daily over a period of 6 months, Brose et al [39] found poorer working memory performance on days with greater negative affect. Conversely, on days when positive affect was higher, working memory performance was improved, which also related to higher task-related motivation [40]. In line with these findings, Sliwinski et al [41] identified a within-person association between higher daily stress ratings and slowed response time on a working memory task; this effect was particularly pronounced in older adults. However, one of the first studies to use repeat cognitive assessment of attention with a reaction time task using palm pilots (prompted 100 times over 7 days) found no association between momentary affect ratings and reaction times [36].

Taken together, there is evidence from the existing literature that suggests negative affect may reduce working memory and semantic recall abilities, at least in controlled settings. However, considerable heterogeneity in the main effects and interaction effects suggests that there is still much to be learned about the role of affect in cognitive performance, particularly when examined outside of the laboratory context and in clinical populations where affect dysregulation may be a primary symptom. In this new digital era, the consistency with which people carry smartphones could be useful in allowing for quick, momentary probes about an individual’s current affect as it changes in daily life. Such assessments of affect could then be paired with smartphone cognitive assessments of different types (eg, attention, working memory, and recall) to provide a more detailed picture of how people’s affect varies with task performance.

#### Motivation

Motivation is thought to drive the allocation of effort, which in turn affects cognitive performance [52]. Measuring the influence of motivation has long been a core feature of clinical neuropsychological assessments with the inclusion of measures designed to assess effort and engagement as checks for validity in testing [53]. However, although these standard measures of effort capture motivation in a given moment in a laboratory or clinic setting, in daily life, motivation varies and depends on factors such as affect, the given rewards or costs of a task, and practice. Ellis et al [38] theorized that strong affect decreased performance by diverting attentional resources to the affective experience versus the task at hand. Adding nuance to this explanation, Pessoa [54] proposed a dual-competition framework of affect, motivation, and executive control. In this framework, both affect and motivation are hypothesized to enhance or impair executive control depending on whether the emotion or locus of reward is aligned or divergent from the task objective. This conceptual model was exemplified through a study by another group and showed how the modulation of reward potential (ie, money) on each trial was positively correlated with both behavioral response time and blood oxygen level–dependent response (ie, functional magnetic resonance imaging–measured neural activation), with the hypothesized mechanism being increased motivation to perform at one’s best when there is a relatively high payout [42]. Another factor underlying motivation’s influence on cognitive performance is practice or task familiarity [43]. When accuracy was measured across 30 trials of a complex multistep judgment task, a person’s self-reported motivation had a greater positive effect on performance in later trials. In other words, when a task is well learned, differences in motivation level have more of an effect on accuracy, compared with when a task is unfamiliar [43]. One study examining self-reported motivation and cognitive performance serially over 100 days found that higher daily motivation ratings were positively correlated with higher scores on verbal and spatial working memory tasks [40].

Although daily retrospective self-reports of motivation have been assessed in relation to working memory [39,40], what remains unknown is the extent to which naturally occurring fluctuations in state-based motivation throughout a given day might influence performance on tasks of working memory or other cognitive domains (eg, processing speed and recall). Using smartphone assessment, one could probe for real-time self-reported motivation before or after a test of cognitive function. Alternatively, tests could include passive or built-in measures of effort that determine the level of engagement in a given task. Simultaneously, sensing metrics, such as time of day and GPS location, could be used to determine the contextual factors that correlate with task engagement. Finally, motivation and cognition could be better studied in tandem via smartphone assessment through the gamification of cognitive tasks that are modeled after smartphone games that participants may have prior familiarity with. In contrast to traditional tests of cognition that provide no immediate reward for the patient or participant, smartphone tasks can easily incorporate point systems, potentially tied to monetary or other rewards, which could allow for observational or experimentally induced modulation of task engagement.

### External Contextual Modifiers of Cognitive Performance

In this section, we have selected studies that measure the influence that factors, outside of the person, have on cognitive performance in various domains, including attention, working memory, executive function, and memory. We will describe studies that seek to understand the impact of a person’s environment, specifically the time of day (morning, afternoon, and evening), the quality and amplitude of environmental noise, and the impact of recent social and physical activity on individuals’ task performance. The studies included in this section are also listed in Table 1.

#### Time of Day

Internal circadian rhythms, driven by the time of day, have been shown to impact cognitive performance. One study of healthy participants aged 10-12 years found that time of day impacted performance on challenging trials of visual working memory and processing speed tasks [44]. Another study measured working memory in the morning and at night across multiple days, in both younger and older adults. Rather than time of day, working memory performance varied with self-reported alertness, which was higher for younger adults in the evening and older adults in the morning [46]. Similarly, a study of young adults found that errors on a task of sustained attention increased with self-reported sleepiness [45]. A review of circadian rhythms and cognitive performance suggested that time of day had a significant effect on a wide range of cognitive tasks, including those of attention, executive functioning, and memory. Notably, performance fluctuation was linked to individual differences in peak circadian arousal [55]. In summary, this literature suggests there is a relation between cognitive function and time of day; however, this relationship seems to rely mostly on time of day’s connection to intraindividual alertness. Mobile assessment can be particularly useful in relating the time of day to cognition using time stamps captured with assessments that can be scheduled at specific times or in random intervals throughout the course of a day. Furthermore, subjective alertness could be captured through momentary reports of wakefulness at the time of the cognitive assessment. Eventually, passive measurement of alertness may be collected using touch screen latencies when typing or through smartwatch sensors that measure heart rate and other biometrics.

#### Surrounding Noise

Distractions are inherent in a noisy or chaotic environment and intuitively impact cognitive performance. In the literature on auditory distractions, the primary area of study has been in the domain of working memory, where a series of findings suggests that the irregularity of sound is most impairing to cognitive performance, rather than the absolute volume of the noise in the environment [56]. Of all types of cognitive tasks, auditory working memory tasks involving maintenance of a series or order of information appear to be most affected by auditory distraction [56]. Similar to the proposed mechanism for affect and motivation, noise is thought to impair performance when attention is pulled away from the task at hand and toward task-irrelevant stimuli, particularly an irregular nonhabituated stimulus [57]. One study demonstrated a particularly large impact of sound on attentional abilities when the distractor and task were similar (eg, auditory-verbal), as this requires more resources for differentiation and suppression than when the task is more unique from the distractor (eg, visuospatial) [47]. Another study found that although distracting ambient noise did not affect objective performance on working memory and reasoning tasks, the addition of environmental noise was related to significantly higher subjective stress ratings and greater perceived difficulty of a given task [49]. Although most studies of noise and cognitive performance have used younger adults in experiments, there has been some investigation into age as a moderator of the influence of irrelevant noise on working memory [48]. When recorded ambient office-noise was played during a visual working memory task, there was a similar level of impairment for both younger and older adults [48]. All the studies above experimentally constructed a *noise condition* to study its effect on cognitive performance, and yet, noise is a product of unpredictable real-world environments that can be challenging to replicate in laboratory settings. Here, mobile assessment could allow for a real-time capture of ambient noise via the use of a phone’s microphone or real-time self-reports of the characteristics of a given environment. Moving forward, sensing technology using advanced data analytics (eg, machine learning algorithms for sound detection) could be helpful in determining the type of audio in a given environment, for example, whether the sounds are of a noisy subway train or a conversation. These categories of sound could then be classified, measured in duration, and linked to cognitive function outcomes, providing individualized assessment results in the context of a person’s unique set of daily environments.

### Recent Physical and Social Activity

The frequency and recency of physical and social activities appear to impact intraindividual variability in cognitive performance. For example, in a study of healthy adults, those with lower baseline working memory capacity performed significantly better on working memory tasks when they engaged in 30 min of cardiovascular exercise immediately before the tasks [58]. Furthermore, in an 8‑day daily diary study in young, middle-aged, and older adults, older adults reported fewer memory failures on the day of and day after physical activity; there was no effect of physical activity in younger adults [50]. In another study examining relationships between physical activity and cognitive performance in older adults, no significant correlation was found between same-day or day-to-day average physical activity and cognition. However, physical activity did explain significant variance in within-person performance in certain cognitive domains, most notably processing speed [51]. Furthermore, in mental illnesses that largely affect cognition, such as schizophrenia, a recent meta-analysis showed that aerobic exercise in these patients was associated with improved attention and working memory [59]. Using smartphone assessment, physical activity can be measured objectively and unobtrusively via an accelerometer. This passive measurement could be enhanced with heart rate detection in associated devices such as smartwatches. Recording physical activity using mobile devices has the potential to add precision to our understanding of how duration, intensity, and recency of physical activity may influence subsequent cognitive performance.

Few studies have looked at the impact of one’s recent recreational or social activities on moment-to-moment cognitive performance, and existing studies are generally specific to samples of healthy older adults. In one study, 60 older adults were prompted via personal digital assistant (PDA) 5 times per day for 1 week to answer questions about their location and to choose a category of their most recent activity. In addition, 2 out of these 5 daily assessments included a measure of semantic reasoning, where an overall category was selected in relation to a list of words. Results indicated that when participants reported having recently engaged in *intellectual activities* (eg, reading and crossword puzzles), scores on the semantic reasoning task were significantly higher [32]. A 7-day study of healthy older adults examined short-term associations between a number of daily activities and performance on web-based tests of processing speed, memory, and reasoning. Intellectual activities were not significantly correlated with cognitive scores, as seen in the study described above; instead, same-day individual or small group social interaction was associated with higher memory scores and response times. Of note, recent physical activity in this study was not associated with better cognitive performance [35]. These findings suggest a need to further explore the mechanisms by which recent social interaction improves cognitive performance and whether or not this finding is limited to older adults or to other specific individual differences.

Social activity could be measured through subjective reports of recent or current activity from smartphone assessments administered throughout the day. Furthermore, the identification of activities could be enhanced and less burdensome through GPS tagging of specific centers of activity such as school, work, friends’ houses, or other locations where socialization takes place (eg, church, gym, and restaurants). Finally, smartphone assessments of cognition can be paired not only with identification of recent activity but also with important subjective ratings such as how important or enjoyable a person found their most recent activity, as this may be even more essential to cognitive performance than the activity type itself.

### Limitations of Research on Modifiers of Cognitive Performance

In summary, research to date suggests that both internal (eg, affect and motivation) and external (eg, time of day, surrounding noise, and recent activity) variables can impact cognitive performance at any given moment. Although this research has shed light on the question of what gives rise to real-life variability in cognitive function, many of the abovementioned studies were limited by traditional methods of data collection. For example, testing cognition in a laboratory or clinical setting, across several time points, ignores the influence of one’s real-world environment, activity levels, and social engagement. Furthermore, performance was often measured in one cognitive domain or in one age group, which does not allow us to extrapolate results to other cognitive constructs and to people across the life span. Finally, some studies relied on retrospective reports of contextual factors or subjective cognition, which are vulnerable to biases. To better understand the unique impact that potential internal and external modifying factors can have on intraindividual variability in cognition over time, we will need to improve the quality and ecological validity of our assessments, including gathering data at a higher temporal frequency and in real time.

### Studying Intraindividual Variability in Cognition via Mobile Technology: Current Evidence

From the existing body of research, it is clear that internal and external factors of one’s environment contribute to cognitive performance. Furthermore, a number of the studies described above suggest that individual differences play a role in determining which, and to what extent, contextual factors affect cognitive performance. Nonetheless, relatively few studies have used repeated measurements across days or weeks to establish temporal relationships between cognitive performance and internal and external modifiers of cognition. Limited research in this area is, at least in part, due to constraints accompanying traditional laboratory or clinic-based research designs. By employing repeated measurements via mobile assessment, there is an opportunity to better understand how cognition and its modifiers temporally covary in real-world environments.

Building off research using ESM and EMA [12,60], mobile devices have been increasingly used in mental health assessment. Smartphone and tablet apps have the capacity to capture a person’s unique fingerprint of response in different domains, ranging from establishing new behavioral habits to symptoms of severe mental illness or progressive neurological disorders. Mobile app–based assessments allow researchers and clinicians to reach people who face barriers to participating in laboratory-based studies or attending regular mental health care appointments [61]. One of the greatest benefits to using mobile devices, specifically smartphones, for ambulatory assessment is capitalizing on their ubiquity: owners carry them on-person most of the time and check them up to hundreds of times in a given day [62].

The incorporation of smartphone-based sensors, such as accelerometers, GPS, and microphones, is a new application of digital assessment that specifically allows for passive measurement of a person’s real-world context and environment. For example, accelerometers capture a proxy for physical activity, which can then be characterized into activity types (sedentary, walking, and running) and correlated with metrics of cognitive variability. By identifying the patterns within a person’s location data from GPS, we can observe how people interact with and traverse their environments through metrics such as distance and visit frequency over time. By taking advantage of a phone’s built-in microphone, there is a potential to better understand the environmental context in which a person is thinking and behaving from moment to moment.

### Mobile Assessment in Neuropsychology

**Figure 1:**
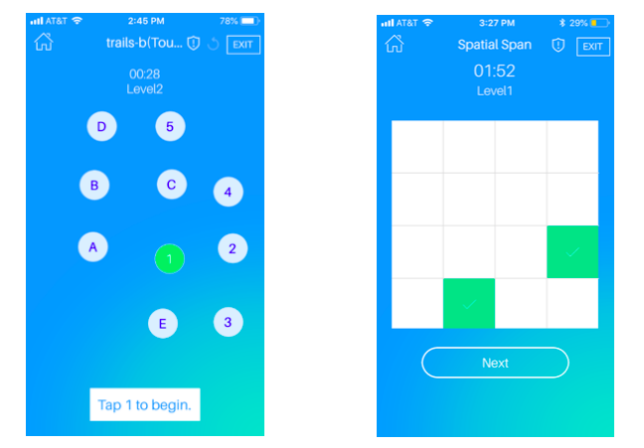
Digital equivalents of traditional neuropsychological tests. In the mindLAMP app, the traditional Trailmaking Test B is translated to the smartphone screen (left). The task measures the accuracy and speed of finger taps between alternating numbers and letters. Spatial Span as an analog task involves a physical board with cubes; on a smartphone, squares light up in a sequential order, followed by a blank grid where the participant taps the same sequence of squares previously shown.

In the field of neuropsychology, traditional pencil and paper tests have only just begun to be translated to computerized versions of these tests (Figure 1), and utilization rates for computerized assessment remain below 10% [63]. A review of studies comparing computerized neuropsychological tests with their analog versions found reliability was similar if not improved on computerized versions. However, normative data for pencil and paper tests could not be used interchangeably with the digital versions, given the smaller sample size for digital tests and differences in the sample characteristics [21].

Beyond replicating existing tests, computerized versions of neuropsychological tests have yet to capitalize on additional passive or process-based data that are possible with mobile technology. For example, response latencies and adaptive testing methods can be used to identify a precise and unique range of within-person cognitive performance. To date, real-time cognitive data collection via ESM and EMA has yet to be fully embraced by the field of neuropsychology, but it has the potential to inform diagnosis and intervention efforts by capturing patients’ fluctuation in cognition [64]. Although digital cognitive assessment is growing exponentially with the use of computers and mobile devices, some have argued that the field of neuropsychology is in the midst of *a technology crisis*, and they suggest that high-frequency data capture is one of the key methods the field should be leveraging [65-67].

A review by Moore et al [68] identified 12 studies that used self-administered mobile cognitive assessments ranging from 1 to 5 times per day over 1-14 days, across various age groups and populations. This review reported high adherence rates (80% on average) and strong construct validity, mostly in the domains of attention, working memory, and executive functioning. Notably, very few of these studies reviewed combined smartphone-based cognitive assessment with real-time measurement of other temporally dynamic contextual variables, and a number of studies only sampled cognition 1 time per day or less. For this methodology to assess potential links between variability in cognitive performance as it relates to context and state variables, a high frequency of measurement is key.

### Review of High-Frequency Measurement Mobile Assessment Studies

To highlight the existing literature that has deployed high-frequency assessment, 14 studies were selected that used mobile paradigm-based assessments of cognition at a frequency of 2 or more times per day for at least one week (2). The majority of studies included in this study were conducted between 2014 and 2019. Out of the 14 studies, 6 included a nonclinical sample of young or middle-aged adults, 2 centered specifically on healthy older adults, and 1 in children. A total of 5 of the 14 studies included samples of substance users or those at risk of smoking or alcohol abuse. Others featured clinical samples, including adults with depression and Parkinson disease. Eight out of the 14 studies, typically the more recent, used a smartphone for mobile assessment, and 5 of the 14 studies used a PDA device, one used a Nokia flip phone, and the other used a smartwatch. The majority of studies used working memory paradigms as the primary cognitive outcome measure, although other cognitive domains such as verbal and visual memory, attention, processing speed, and motor speed were also measured alone or alongside working memory in these studies. Of the 14 studies, 12 deployed repeat mobile tests for 1‑2 weeks; other durations included 4, 6, and 24 weeks. A total of 6 studies prompted cognitive assessments 2-3 times per day, 6 studies prompted 4-6 times per day, and 2 studies prompted at an even higher frequency. In addition to cognitive performance, 4 out of 14 studies reported data collection related to momentary affect or mood, 3 out of 14 studies recorded reports of recent activity types or social settings, and 3 out of 14 studies examined time of day or fatigue in relation to cognitive performance. Generally, studies found high levels of concordant validity between mobile cognitive assessments and traditional in-laboratory measures.

Approximately half of the studies mentioned in Table 2 examined contextual or internal state-based variables in relation to cognitive performance; their findings on the association between context and cognition were mixed. Smartphone semantic reasoning and memory scores were greater after recent intellectually stimulating activity [32], slower smartphone-recorded reaction times were associated with greater mental fatigue [69], and increased error rates were seen in phone-based working memory and attention [70]. On the other hand, 2 studies conducted almost 15 years apart found no within-person associations between momentary mood ratings and working memory [71] and reaction time performance [36]. It should be noted that studies of repeat mobile cognitive assessment have used a variety of mobile platforms, different populations, and different ways of measuring the same contextual variables (eg, scales or dimensions of mood); as such, findings require replication using standardized methods.

Several studies commented on the psychometrics of reliable within-person fluctuations and the need to understand the drivers of these patterns of fluctuations. Dirk and Schmiedek [33] examined the psychometrics of repeated mobile assessment of cognition in children and found that moment-to-moment and day-to-day performance had reliable amounts of intraindividual variability, which was indicative of individual differences and patterns of response. Furthermore, more recent work by Sliwinski et al [15] suggested that within-person fluctuation in processing speed and working memory across the course of a day was reflective of meaningful, but unknown, contextual moderators of the intraindividual variability observed. This indicates the need to explore and identify the unknown contextual variables that influence variability in within-person cognitive functioning. Furthermore, nomothetic approaches to analyzing within-person contextually based modifiers of cognition may fail to produce reliable or meaningful findings, as the influence of particular contextual factors (eg, time of day) may be dependent on individual differences. In addition to statistical analysis that uses aggregate measures, idiographic approaches [78] could be used to understand the temporal dynamics between context, state, and cognitive function of a unique individual, which may be reliably different from another individual. Through the use of idiographic analyses such as individualized time-lagged modeling and network analyses, there is an opportunity to develop both personally targeted and ecologically generalizable interventions [31].

**Table 2:** Mobile assessment of cognition.

Reference	Sample	Assessment tool	Length (weeks)	Daily frequency	Cognitive domain	State or context variables	Result
Allard et al [32]	60 older adults	PDA^a^	1	2 times/day	Semantic reasoning and memory	Location, social setting, and recent activities or behaviors	Cognitive performance improved following intellectually stimulating activities
Cormack et al [71]	30 adults with depression	Apple watch	6	3 times/day	Working memory	Mood	High adherence, moderate concordance, and no relationship between momentary mood and cognition trajectories
Dagum [72]	27 young adults	Smartphone	1	Continuous	Working memory, executive function, and languages	Not specified	Digital biomarkers (eg, taps and swipes) highly correlated with traditional in-laboratory neuropsychological test scores
Dirk and Schmiedek [33]	110 participants aged 8-11 years	Smartphone	4	3 times/day	Working memory	Motivation, affect, sleep, and physical activity or accelerometer	Greater working memory variability measured by phone correlated with lower performance on in-laboratory cognitive and academic tests
Lipmeister et al [73]	44 patients with Parkinson disease; 35 controls	Smartphone	24	6 times/day	Motor speed	Motor symptoms	Phone tests of motor speed correlated with questionnaire measures and differentiated patients from controls
Pal et al [74]	12 meth addicts; 20 controls	Laboratory computer and smartphone	2	2 times/day	Working memory	Not specified	N-Back and Stop Signal on iPhone correlated with laboratory-based tests; speech detection on Stroop task did not work; no between-group differences
Price et al [69]	21 young adults	Smartphone	2	3 times/day	Working memory, attention, and processing speed	Mental fatigue	Fatigue ratings positively correlated with longer reaction times on attention task
Salthouse and Berish [36]	420 adults	PDA	1	15 times/day	Reaction time	Time of day and mood	Large within-person variability; no significant relation between time of probe or mood and reaction time
Sliwinski et al [15]	219 adults	Smartphone	2	5 times/day	Processing speed and working memory tasks	Not specified	High construct validity, reliability, and within-person variance
Schweitzer et al [75]	114 older adults	Smartphone	1	5 times/day	Memory and executive function	Physical environment and social interaction	High adherence and concordance with traditional neuropsychological test scores
Schuster et al [76]	39 high-risk smoker young adults	PDA	1	5-7 times/day	Working memory	Not specified	High feasibility or compliance and construct validity
Tiplady et al [70]	38 adults who frequently consumed alcohol	Cell phone	2	2 times/day	Attention and working memory tasks	Alcohol consumption	Greater errors on phone- and laboratory-based tasks after alcohol consumption
Waters et al [77]	22 smokers; 22 controls	PDA	1	4 times/day	Working memory	State anxiety	High adherence and high reliability
Waters et al [34]	119 smokers	PDA	1	4 times/day	Attentional bias	Not specified	Between-subject craving and laboratory attentional bias associated with PDA Stroop attentional bias

^a^PDA: personal digital assistant.

### Implementation and Future Directions

Although new innovative forms of smartphone assessment are becoming increasingly common, there are several likely reasons for the dearth of research, to date, using mobile technology to study cognition and context. The first is the financial barrier to developing app-based assessment tools; in addition to this, there are significant time demands and logistical challenges of establishing the feasibility, reliability, and validity of these new instruments. In traditional neuropsychological assessment, the objective has been to measure cognitive function in a quiet setting with few distractions to obtain the best possible performance. However, when measuring cognitive function with a mobile device, there is an opportunity for ample variability and distractions. This contextual *noise* can, in fact, be a strength, reflecting a more ecologically valid environment. However, to form an accurate picture of a person’s functioning across contexts, a large volume of data is needed. Analysis of acquired mobile assessment data will need to take into account the high frequency of measurements within individuals and between groups to produce clinically useful normative comparison data [79]. This brings forth the challenge of localizing the driving factors of within-person variance while also quantifying practice effects. Some have raised concerns about the use of computerized neuropsychological assessment due to the lack of standardization and normative data, which when applied to patient care could ultimately lead to poor clinical decisions and errors in diagnosis. There have been several calls to the field to address this concern by focusing on resources and investigating the psychometrics of mobile cognitive assessment [29,61,79] and centralizing the development of a toolbox of standardized mobile neuropsychological assessments that can be validated more efficiently through a network of clinical researchers across the field [80].

**Figure 2:**
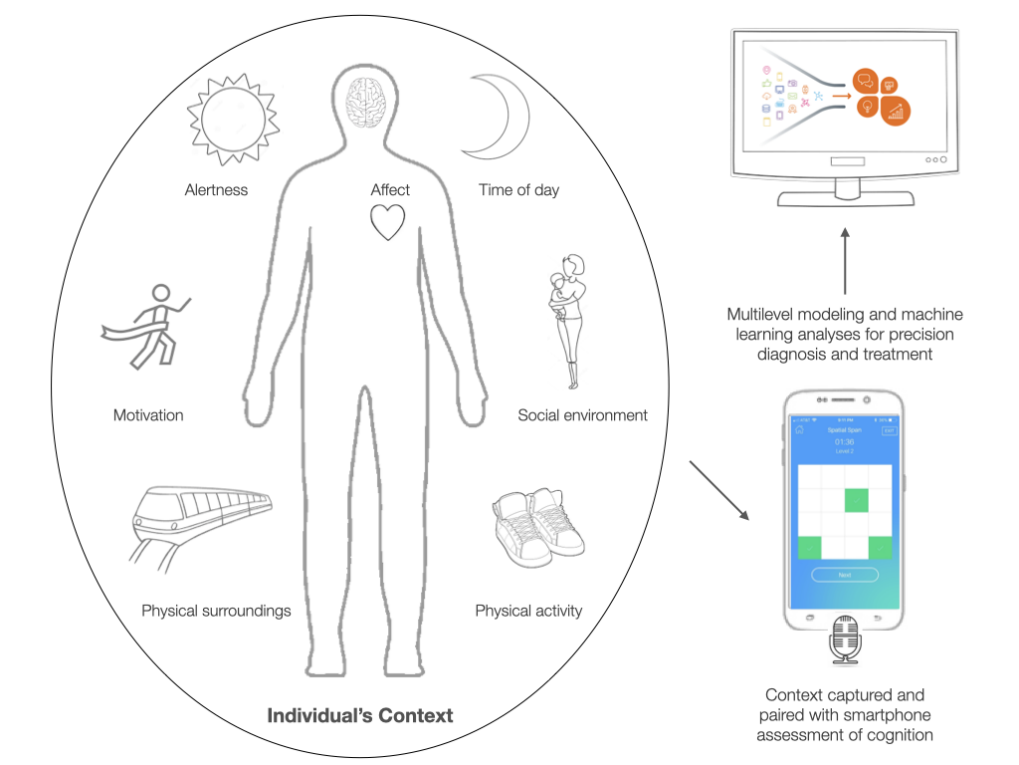
Model of mobile assessment of intraindividual variability in cognition. Internal state-driven variables include affect motivation and alertness. External contextual variables include time of day, social environment, physical surroundings, and physical activity. Taken together, these factors give rise to fluctuations in cognitive performance. This can be captured in real time using a game-like smartphone assessment of cognition alongside sensing tools such as a smartphone microphone and GPS, which seamlessly capture information about one’s environment. Advanced statistical methods can be used to analyze data to find patterns of intraindividual variability in cognition in real-world contexts.

Inherent in pioneering mobile health technology is the challenge of developing measurement tools that are usable and study designs that are informative. However, unlike ever before, there is immense potential to study familiar neuropsychological constructs outside the laboratory setting and in the environments in which they naturally operate. To move beyond proof-of-concept validation of ambulatory assessments, the field is faced with the challenge of creating new paradigms that are not mere replications of existing neuropsychological measures but are designed to be independently administered across time, contexts, and devices. For example, moving beyond the gold standard neuropsychological measures of cognition, some studies have explored how smartphone activity, such as swipes and taps, could serve as a proxy for working memory, episodic memory, executive function, language, and intelligence in a pilot study of healthy adults [72]. The accessibility and availability of smartphones sets the stage for the emergence of new scalable research efforts that could easily recruit participants from both healthy and clinical samples and record vast quantities of new data. The results shown in Figure 2 are the ways in which some of the key ecologically situated variables can be measured using smartphones and connected to digital neuropsychological tasks performed on digital devices. The momentary and state-related variables can be measured passively (eg, GPS and microphone) or actively (eg, surveys). Together, data streams can be combined and analyzed using computational and machine learning methods to identify relationships between cognitive performance and contextual momentary factors.

Ultimately, the goal of mobile assessment methods is to refine the understanding of clinical impairments in cognition and give rise to preventative interventions for mitigating cognitive dysfunction and decline. New work is being done to develop a smartphone app designed to improve clinical care based on patients’ and clinicians’, in addition to researchers’, needs. The objective of this app development is to create an open platform that can be used across a variety of research and clinical settings [18]. Unlike other existing mobile health apps developed for a specific population or function, the mindLAMP (Learn, Assess, Manage, Prevent) has been created to allow for customizable surveys, sensors, cognitive tests, and schedules of notification-prompted assessment. The app seeks to integrate active assessments such as surveys and cognitive games with passive sensing data (eg, GPS, pedometer, and microphone) and even phone metadata, such as the number of times other social media or communication apps are used on the phone. With the rich dimensionality of smartphone data from apps such as these, multilevel modeling and machine learning analyses would be logical directions to take analysis to elucidate the causal links and predictive relationships between cognitive performance and state-based and environmental factors.

Like any new method, repeat assessment of cognition and context via mobile technology must be carefully employed. Given the complexity and sheer data volume of temporarily dense, longitudinal, and dynamic smartphone and sensor data, it will be important for theory-driven hypotheses to guide this work instead of only data-driven models that will likely identify spurious correlations. Moreover, given the nature of these new data, care must be taken to ensure its privacy protections and ethical uses [81]. Looking back at the history of genetics and neuroimaging, it is clear that the greatest progress with this new spade will emerge from interdisciplinary collaborations. As health care moves toward personalized medicine, the field also has an opportunity to move toward personalized assessment of cognition. Although population-level screening tests and individualized in-office neuropsychological evaluations will remain important, they cannot fully capture the social, physical, and environmental variance that each individual experiences. Fortunately, mobile technologies such as smartphones can collect such data and thus present the opportunity for a paradigm shift with personal devices capturing personal measurements to deliver personal cognitive profiles and treatment directions.

## Abbreviations

EMA: ecological momentary assessment
ESM: experience sampling method
PDA: personal digital assistant
